# PReS-FINAL-1017: Antioxidant superoxide dismutase activity is elevated in jia but not associated with disease activity

**DOI:** 10.1186/1546-0096-11-S2-P14

**Published:** 2013-12-05

**Authors:** A Radziszewska, C Rowden, L Suffield, S Ursu, L Kassoumeri, LR Wedderburn, Y Ioannou

**Affiliations:** 1 Arthritis Research UK Centre for Adolescent Rheumatology at University College London, Great Ormond Street Hospital and UCLH, University College London, London, UK

## Introduction

Oxidative stress has been implicated in the pathogenesis of a number of autoimmune diseases including rheumatoid arthritis. The antioxidant superoxide dismutase (SOD) is one of the most important enzymes involved in combating oxidative stress. It exerts its protective effect by neutralizing free radicals to form oxygen and hydrogen peroxide.

## Objectives

The aim of the present study was to measure SOD activity in juvenile idiopathic arthritis (JIA) patients and to assess whether these levels are influenced by disease activity or JIA subtype.

## Methods

Serum SOD activity was measured in 166 pediatric and adolescent JIA patients attending Rheumatology clinics at Great Ormond Street Hospital and University College London Hospital and in 17 healthy controls (HC) using a commercial colorimetric assay. Median age at sampling was 16.37 [IQR 10.41-18.04] years for JIA patients and 14.78 [IQR 10.22-23.03] for HC). Median age of JIA onset was 5.68 years [IQR 2.30-11.23]. Female:male ratio was 95:71 (57.2% female) in JIA patients and 11:5 (64.7% female) in HC. Similar numbers of patients for each subtype were included (oligoarticular n = 24, extended oligoarticular n = 27, polyarticular n = 37, systemic-onset n = 42, enthesitis-related n = 33) with the exception of psoriatic arthritis (n = 3). Disease activity was defined by presence of swollen joints and an erythrocyte sedimentation rate (ESR) greater or equal to 20 mm/hr. Inactive patients were those with no swollen joints and an ESR less than 20 mm/hr. Patients who did not fulfill both criteria were excluded from analysis relating to disease activity.

## Results

SOD activity was shown to be elevated in JIA patients (median = 6.38 U/ml [IQR 4.83-8.70]) compared to controls (median = 4.03 U/ml [IQR 2.18-6.48]) across all subtypes (*p *= 0.0009). There was no statistically significant difference in SOD activity between subtypes. No difference in SOD activity was observed between patients with active disease and those with inactive disease and SOD activity did not change depending on drug treatment. However, SOD activity did change with anti-nuclear antigen (ANA) status, with ANA positive patients having elevated SOD levels (median = 7.22 U/ml [IQR 5.08-9.64]) compared to those who were ANA negative (median = 5.95 U/ml [IQR 4.63-7.98]) (p = 0.022).

## Conclusion

In conclusion, elevated activity of the antioxidant SOD may be a response to increased oxidative stress in JIA. However, it does not seemingly represent a useful biomarker of disease activity. The association with ANA is interesting and we are currently investigating whether SOD levels correlate with uveitis.

## Disclosure of interest

None declared.

